# Germline RAD54L with somatic POLE defect implicated in Hypermutation phenotype: case report

**DOI:** 10.1186/s12876-020-01403-y

**Published:** 2020-08-05

**Authors:** Bisan Abdalfatah Zohud, Meiling Wang, Xin Cai

**Affiliations:** Department of Oncology, The First Affiliated Hospital of Dalian Medical University, Dalian, Liaoning 116000 China

**Keywords:** Hypermutated CRC, POLE, Rad45L, Homogenous recombination

## Abstract

**Background:**

Colorectal cancer is one of the most frequent causes of death among cancer patients. Hypermutated CRC is an extraordinary case of cancer, but curable if detected at early stages. However, the mechanism for developing a hypermutated CRC remains unclear. An association between RAD54L germline mutation and POLE exonuclease domain hypermutated cancer has not been reported before.

**Case presentation:**

We present a rare case of a 41-year-old Chinese female with a right-sided colon adenocarcinoma who harboured a (p.P286R) POLE somatic mutation. Genomic analysis was performed using the Illumina HiSeq Sequencing platform, which, identified MSS tumour with a (c.1093_1169 + 15dup) germline mutation in RAD54L gene and tumour mutation burden of 377.0 Muts/Mb. Based on our report a new mechanism for developing hypermutated colon cancer has been conjectured through a novel RAD54L_POLE DSBR pathway.

**Conclusion:**

This report highlights the clinical importance of next-generation sequencing technology in diagnosing rare tumours and investigating novel mechanisms for developing exceptional genetic diseases.

## Background

Colorectal cancer (CRC) is one of the most frequent causes of death among cancer patients [1]. Different subtypes have been classified based on the molecular changes that occur in colon cancer. Hypermutated colon cancer was defined as a tumour with a somatic mutation rate > 12 per megabases and had a median of 728 non-silent mutations [2]. Sixteen percent of CRCs are hypermutated according to The Cancer Genome Atlas Network [3]. Diverse causes of hypermutation were identified depending on cancer type. The main causes of the mutations that accumulate in hypermutated CRC are mismatch repair (MMR) defects and somatic alterations in the exonuclease domain of DNA polymerase epsilon (POLE) [4].

A POLE colorectal tumour tends to be microsatellite stable (MSS), occur during early-onset cancer (before 50 years age) and be located predominantly in the right colon [5].

Here, we present an exceptional case of a hypermutated colon cancer patient with a POLE P286R mutation who harboured a germline mutation in the RAD54L gene. Based on our report, the relationship between RAD54L mutation and hypermutation has not been reported previously.

We hypothesized a novel mechanism for developing hypermutated colon cancer.

## Case presentation

**Fig. 1:**
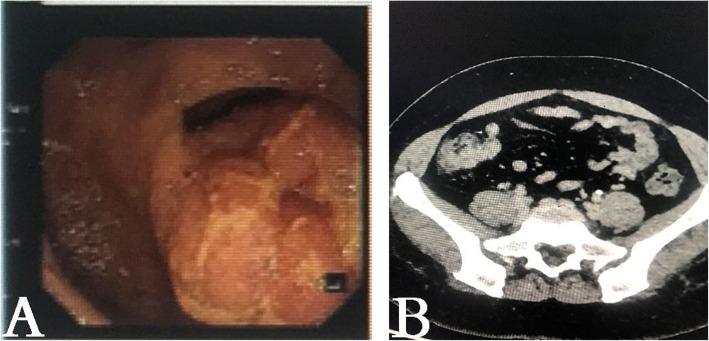
**a** Colonoscopy shows a protuberant mass, size 2.5 cm, in the caecum. **b** Abdomen CT scan: the delayed phase of contrast-enhanced CT showed localized intestinal wall thickness in the caecum and unclear serosa layer. No lymph nodes were noted

We report a rare case of a 41-year-old Chinese woman with right-sided colon adenocarcinoma. Her medical history showed no obvious abnormality. No family history of colon cancer was reported. She was asymptomatic when a colonoscopy confirmed a mass in the caecum (Fig. 1a). A CT scan of the abdomen showed caecum wall thickness (Fig. 1b). No evidence of liver or lung involvement was noted.

**Fig. 2:**
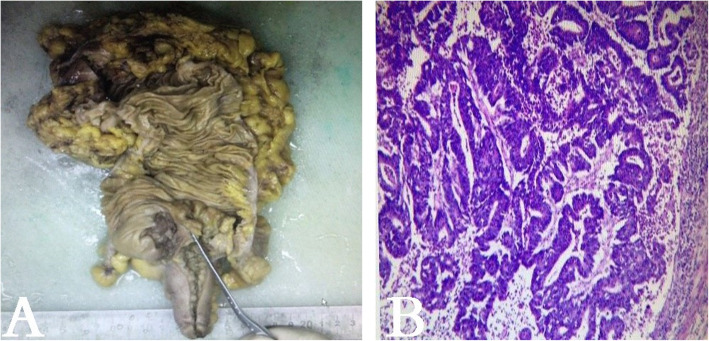
**a** The gross specimen: a neoplastic mass in the ilea-caecum. **b** Hematoxylin and eosin staining confirmed an invasive moderately differentiated adenocarcinoma of the colon with deep muscular layer invasion and intravascular tumour thrombus and no definite nerve invasion

**Fig. 3:**
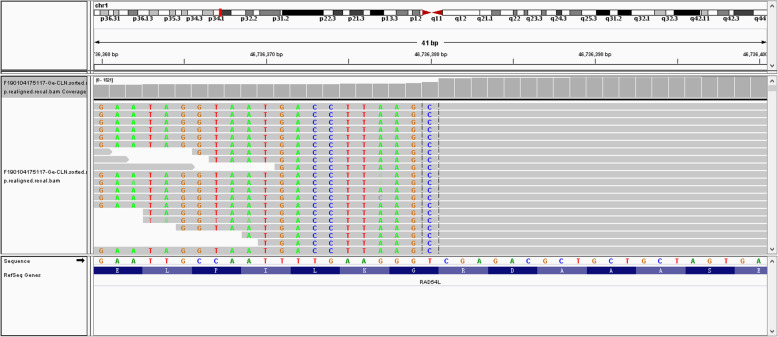
Sequencing reads of RAD54L were visualized by the Integrative Genomics Viewer (IGV). Genomic coordinates (chromosome = “chr1”, start = 46,736,380, end = 46,736,381)

In 2018, a right-sided colectomy was performed (Fig. 2a). Histopathological examination revealed a moderately differentiated adenocarcinoma with lymphovascular invasion (Fig. 2b). Immunohistochemistry tests for MLH1, MSH2, MSH6, PMS2, CD31, CD34 and D2–40 were positive. No mutations were detected in the KRAS, NRAS and BRAF genes. Genomic analysis was performed using the Illumina HiSeq Sequencing platform, which covered the complete exonic regions of the 425 gene, a selected set of intronic regions associated with chromosome rearrangement, regions affecting mRNA alternative splicing, and specific genomic loci associated with microsatellite instability (MSI). This test evaluates all of the mutation types, the tumour mutation burden (TMB), the status of the mismatch repair (MMR)-related genes, microsatellite (MS) status, and inheritance risk of the disease genes within the patient. We have tested not only the tumour tissue but also the blood samples of the patient. Blood DNA is the most commonly used sample for the detection of germline mutations in patients with non-haematological diseases. Germline mutations and tumour-specific mutations screened in comparison with blood negative controls. The test results revealed that her tumour was MSS with a (c.1093_1169 + 15dup) RAD54L germline mutation (Fig. 3) and a TMB of 377.0 Muts/Mb including ABCB1, AKT1, APC, ATM, BAX, BRCA1, BRCA2, ERBB2, PBRM1, TAP1, TP53, XPC, FGFR1, FGFR4, NF2, CDK8, NTRK2, RAD50, ATR, PIK3R1, SMAD4, SMAD2, POLE and POLH. Since the patient’s parents have passed away, germline mutation was unable to be tested. For humanitarian reasons, we can’t perform genomic analysis test for her child. According to the TNM staging system, she staged as IIA (pT3N0 M0). Subsequently, she was treated with 8 cycles of biweekly FOLFOX (5-FU, leucovorin, and oxaliplatin). Now she is one-year cancer-free.

## Discussion and conclusion

The POLE gene encodes a DNA polymerase, the catalytic subunit of DNA polymerase ε. DNA polymerase epsilon (polε) belongs to the DNA polymerase B family together with polα, polδ, polζ. The catalytic subunit of DNA polymerase epsilon (POLE) is the core of polε, which has two catalytic activities. One is the DNA template polymerase activity, which is responsible for the replication and extension of new DNA chains; the other is the correction activity of the exonuclease region, which is responsible for the recognition and repair of mismatched bases. Among them, the structure of the corrected activity is called the exonuclease domain of POLE (POLE-exo*), which has 3′ → 5′ exonuclease activity, and can recognize and remove errors generated during the replication process. The exonuclease domain mutations of POLE (POLE-EDMs) will result in the loss of correction function, leading to accumulation of mutated genes in cells, resulting in hypermutation. The reported POLE mutation in CRC was p.P286R in the exonuclease domain, showing that the proline amino acid at position 286 has been changed to arginine [4]. The complementary action of the MMR system and POLE proofreading to correct the polymerase errors that occur during DNA replication prevents the accumulation of deleterious mutations. Therefore, any alteration in one system will disrupt the other. Loss of proofreading function emerges earlier than MMR deficiency in hypermutant tumours [2], providing an explanation of associated POLE tumours with MSS phenotype.

Furthermore, numerous germline mutations have been reported in DNA repair pathways, although their contribution to increasing the risk of developing cancer is still unknown. DNA polymerases play a central role in homologous recombination (HR), and different DNA polymerases are involved in DNA synthesis related to homologous recombination repair functions. Homologous recombination (HR) is the main pathway for accurate repair of double strand breaks (DSBs), which is critical for maintaining human genomic integrity. A wide variety of genes are involved in HR, including Rad54, Brca2, Rad52, and Rad51 [6, 7]. The Rad54 paralog is a member of the SNF2/SWI2 protein family, SNF2/SWI2 complex is an ATP-dependent chromatin remodeling complex, that specifically plays an important role in the HR repair pathway of DSB. It promotes chromatin remodeling and maintenance of genomic stability through the HR pathway in concert with RAD51, which is a key protein in HR [8, 9]. Rad54 interacts physically with Rad51 to promote its function in DNA strand exchange, which is a basic step in HR [10]. In yeast, Rad54 is essential for homologous recombination repair based on sister chromatid [11]. Thus, defects in the RAD54 gene lead to reduction in HR efficiency.

Somatic alterations in Rad54L have been identified in non-Hodgkin’s lymphoma, breast and colon cancer [12]. To our knowledge, Rad54L germline mutations have not been reported before in CRC.

In our case, we speculated that RAD54L germline mutations might be one of the hypermutated colon cancer risk events.

The WNT, RTK-RAS, PI3K and TGF-B pathways are often mutated in hypermutated colon cancer. TP53 is one of the most frequently altered genes among non-hypermutated colon tumours [13]. However, our patient case showed TP53 p.R213X exon 6 truncating mutation with 16.6% mutational allelic frequency (MAF). The P53 pathway has been identified to maintain genome stability through control DNA damage checkpoints and modulate several DNA repair systems [14]. Previous studies demonstrated that TP53 modulates the HR pathway and suppresses genome instability through direct interaction with Rad51 and Rad54 proteins [15].

During the HR process, several modes of DNA synthesis are involved in restoring the chromosome integrity, which include various complements of DNA polymerases. POLE is responsible for the synthesis of the second end of the D-loop [16]. On the other hand, in vitro studies demonstrated that Rad54 is vital for displacement loop extension by DNA polymerase. After DNA strand invasion by RAD51 during the HR pathway, RAD54 controls access of DNA polymerase to the invading 3′-OH end [17].

RAD54L plays an important role in the homologous recombination repair pathway. These findings indicate that Rad54L, in cooperation with POLE, maintains chromosomal stability through DNA HR and the P53 signaling pathway; however, their physical interaction is still not defined.

In conclusion, next-generation sequencing is an important tool to unravel genetic alterations associated with rare tumour progression. This article reports for the first time the case of RAD54L germline mutation combined with POLE gene somatic mutation in colorectal cancer. It is speculated that the two have a synergistic effect in hypermutation. The association between RAD54L germline mutation and hypermutated colon cancer has not been previously studied. Thus, this case report suggests a new molecular mechanism for developing hypermutated CRC. We conjecture that a germline mutation in RAD54L may contribute to hypermutated CRC pathogenesis through a novel RAD54L_POLE DSBR pathway.

## Data Availability

Available on request.

## Abbreviations

CRC: Colorectal cancer
MSS: Microsatellite stable
POLE: DNA polymerase epsilon
MMR: Mismatch repair
HR: Homologous recombination
DSBR: Double strand break repair
CT scan: Computed tomography scan
NER: Nucleotide excision repair

## References

[1] Arnold M, Sierra MS, Laversanne M, Soerjomataram I, Jemal A, Bray F (2017). Global patterns and trends in colorectal cancer incidence and mortality. Gut.

[2] Campbell BB, Light N, Fabrizio D, Zatzman M, Fuligni F, de Borja R (2017). Comprehensive analysis of Hypermutation in human Cancer. Cell.

[3] Muller MF, Ibrahim AE, Arends MJ (2016). Molecular pathological classification of colorectal cancer. Virchows Arch.

[4] Briggs S, Tomlinson I (2013). Germline and somatic polymerase epsilon and delta mutations define a new class of hypermutated colorectal and endometrial cancers. J Pathol.

[5] Ahn SM, Ansari AA, Kim J, Kim D, Chun SM, Kim J (2016). The somatic POLE P286R mutation defines a unique subclass of colorectal cancer featuring hypermutation, representing a potential genomic biomarker for immunotherapy. Oncotarget.

[6] van Gent DC, Hoeijmakers JH, Kanaar R (2001). Chromosomal stability and the DNA double-stranded break connection. Nat Rev Genet.

[7] Shrivastav M, De Haro LP, Nickoloff JA (2008). Regulation of DNA double-strand break repair pathway choice. Cell Res.

[8] Mazin AV, Mazina OM, Bugreev DV, Rossi MJ (2010). Rad54, the motor of homologous recombination. DNA Repair (Amst).

[9] Ceballos SJ, Heyer WD (2011). Functions of the Snf2/Swi2 family Rad54 motor protein in homologous recombination. Biochim Biophys Acta.

[10] Heyer WD, Li X, Rolfsmeier M, Zhang XP (2006). Rad54: the Swiss Army knife of homologous recombination?. Nucleic Acids Res.

[11] Clever B, Interthal H, Schmuckli-Maurer J, King J, Sigrist M, Heyer WD (1997). Recombinational repair in yeast: functional interactions between Rad51 and Rad54 proteins. EMBO J.

[12] Matsuda M, Miyagawa K, Takahashi M, Fukuda T, Kataoka T, Asahara T (1999). Mutations in the RAD54 recombination gene in primary cancers. Oncogene.

[13] Cancer Genome Atlas N (2012). Comprehensive molecular characterization of human colon and rectal cancer. Nature.

[14] Williams AB, Schumacher B (2016). p53 in the DNA-damage-repair process. Cold Spring Harb Perspect Med.

[15] Menon V, Povirk L (2014). Involvement of p53 in the repair of DNA double strand breaks: multifaceted roles of p53 in homologous recombination repair (HRR) and non-homologous end joining (NHEJ). Subcell Biochem.

[16] McVey M, Khodaverdian VY, Meyer D, Cerqueira PG, Heyer WD (2016). Eukaryotic DNA polymerases in homologous recombination. Annu Rev Genet.

[17] Li X, Heyer WD (2009). RAD54 controls access to the invading 3′-OH end after RAD51-mediated DNA strand invasion in homologous recombination in Saccharomyces cerevisiae. Nucleic Acids Res.

